# The association between prune belly syndrome and dental anomalies: a case report

**DOI:** 10.1186/1472-6831-12-56

**Published:** 2012-12-18

**Authors:** Maria Daniela Basso, Carla Oliveira Favretto, Robson Frederico Cunha

**Affiliations:** 1 Disciplina de Odontopediatria e Clínica Integrada Infantil, UNIOESTE – Univ. Estadual do Oeste do Paraná, Jardim Universitário, Rua Universitária 2069, Cascavel, PR, (85819-110), Brazil; 2 Departamento de Odontologia Infantil e Social, UNESP – Univ Estadual Paulista, R. Jose Bonifácio 1193, Araçatuba, SP, (16015-050), Brazil; 3 Departamento de Odontologia Infantil e Social, UNESP – Univ Estadual Paulista, R. Jose Bonifácio 1193, Araçatuba, SP (16015-050), Brazil

**Keywords:** Prune belly syndrome, Tooth abnormalities, Child

## Abstract

**Background:**

Prune belly syndrome is a rare condition produced by an early mesodermal defect that causes abdominal abnormalities. However, the literature indicates that disturbances related to ectodermal development may also be present. This is the first case report in the literature to suggest that dental abnormalities are part of the broad spectrum of clinical features of prune belly syndrome. Because the syndrome causes many serious medical problems, early diagnosis of abnormalities involving the primary and permanent dentitions are encouraged.

**Case presentation:**

The authors report the clinical case of a 4-year-old Caucasian boy with prune belly syndrome. In addition to the triad of abdominal muscle deficiency, abnormalities of the gastrointestinal and urinary tracts, and cryptorchidism, a geminated mandibular right central incisor, agenesis of a mandibular permanent left incisor, and congenitally missing primary teeth (namely, the mandibular right and left lateral incisors) were noted.

**Conclusion:**

This original case report about prune belly syndrome highlights the possibility that dental abnormalities are a part of the broad spectrum of clinical features of the syndrome. Therefore, an accurate intra-oral clinical examination and radiographic evaluation are required for patients with this syndrome in order to provide an early diagnosis of abnormalities involving the primary and permanent dentitions.

## Background

Prune belly syndrome (PBS), described for the first time in the 1800s [1], is a rare condition defined by the triad of abdominal muscle deficiency, severe urinary tract abnormality, and cryptorchidism [2-4]. It is caused by urethral obstruction early in development and is the result of massive bladder distention and urinary ascites, leading to degeneration of the abdominal wall musculature and failure of testicular descent. The impaired elimination of urine from the bladder leads to oligohydramnios, pulmonary hypoplasia, and Potter’s facies [5]. The name PBS originated from the wizened, dried-plum appearance of the child [2-4]. Patients with this syndrome also may have congenital cardiac, pulmonary, musculoskeletal, and gastrointestinal anomalies [6,7]. In addition, associations with cleft lip [3], Down syndrome [2], and trisomy of 18 [8] and 13 [9] have been reported.

This syndrome occurs in 1 of 35.000–50.000 live births and most cases (up to 95%) are in males [6]. In another report, the incidence of PBS was 3.8 cases/100,000 live births [10]. Despite advances in care for children with PBS, this condition continues to be associated with high perinatal mortality, which is likely related to the associated prematurity, pulmonary complications [10], and urinary tract malformations [5].

The exact etiology of the generalized disturbance is unknown [4]. According to Straub and Spranger [4], the various manifestations of PBS are produced by an early mesodermal defect. An association was reported between PBS and ectrodactyly-ectodermal dysplasia-clefting [11], which is a rare, autosomal dominant syndrome that is phenotypically characterized by specific abnormalities of the hands, feet, and orofacial region coexisting with ectodermal dysplasia features [12].

Kabakus *et al.*[13] found other abnormalities associated with PBS that support the concept that PBS is caused by an early disturbance of other germ layers as well as mesodermal development. Given these findings, extra-abdominal abnormalities resulting from disturbances of ectodermal and endodermal development should be evaluated in all cases. Although disturbances related to ectodermal and endodermal development may be asymptomatic, an early diagnosis of these disturbances may help to prevent possible future problems [13].

The hypothesis that there may be a combination of PBS to oral problems has been raised. An association was found between this syndrome and isolated gingival fibromatosis and facial dysmorphism [14].

Other reports have shown that congenital kidney and urinary tract anomalies can cause serious renal function problems in the patient, which occasionally progress to chronic renal failure (CRF) and end-stage renal failure (ESRF) [15]. Thus, the patient develops metabolic disorders, such as hyperparathyroidism resulted from CRF or ESFR, which cause secondary oral manifestations [16].

Enamel hypoplasia with generalized hypocalcemic dental lines, demineralization of the trabecular bone of the jaw, loss of the lamina dura, and discontinuity of the mandibular cortical bone were cited as secondary to CRF and hyperparathyroidism [16]. Petechiae, ecchymoses, uremic stomatitis, gingivitis, periodontitis, enamel hypoplasia, and dental pulp obliterations were related to CRF, ESRF, and renal replacement therapy [17-19]. Furthermore, excessive calculus deposits were related to ESRF [15].

This is the first report of a patient with PBS associated with dental anomalies. Given that PBS is a rare disease for which the oral and dental aspects are seldom described in the literature, the goal of the present case report is to contribute to a better understanding of this syndrome.

## Case presentation

The child was included in the Baby Clinic of Araçatuba Dental School program for preventive dental care at 11 months of age, and this clinical case report is being presented when the child is 4 years old.

The child’s mother reported that her child had a PBS during the anamnesis procedure, and he had been undergoing regular medical check-ups since birth because of this finding. The pregnancy was planned by the parents, and the mother received prenatal care. An ultrasound examination performed during the fifth week of gestation showed a gastrointestinal malformation of the fetus.

A subsequent morphologic ultrasound examination revealed the following findings: gastrointestinal abnormality, enlarged kidneys, and a low amount of amniotic liquid. However, although the parents were aware that their child would be born with health problems during the gestation period, they did not receive conclusive information regarding the baby’s condition.

The baby boy was born prematurely at 7 months by caesarian, with a weight of 1450 kg and height of 41 cm. According to the mother, postnatal examination revealed that the baby had a distended and wrinkled abdomen, resembling a dried prune. The form of the penis was abnormal, with an enlarged diameter and lax excess skin. Despite these characteristics, an immediate diagnosis was not made.

Numerous medical examinations were performed during this period, and the results combined with the clinical features resulted in a diagnosis of PBS. The child does not have relatives with any type of syndrome.

These features included abdominal musculature deficiency, gastrointestinal and urinary abnormalities, a larger-than-normal bladder, malformed kidneys (only 1 with normal function), and cryptorchidism (bilateral intra-abdominal testes).

Although only 1 kidney was found to function normally on medical examination, the mother stated that the child did not have problems associated with this and denied impairment of renal function. The mother reported episodes of urinary infection requiring antibiotic treatment that were related to excess skin on the penis in the first to third years of life.

Moreover, the mother reported that the child did not have cardiac or pulmonary disease, and no anal alteration was found. The alterations mentioned above may result from the syndrome.

The characteristics of the inferior and superior limbs, hands, feet, fingers, and toes were normal. Structural abnormalities were only noted in both of the child’s knees, which is a feature consistent with this syndrome, according to the mother.

**Figure 1:**
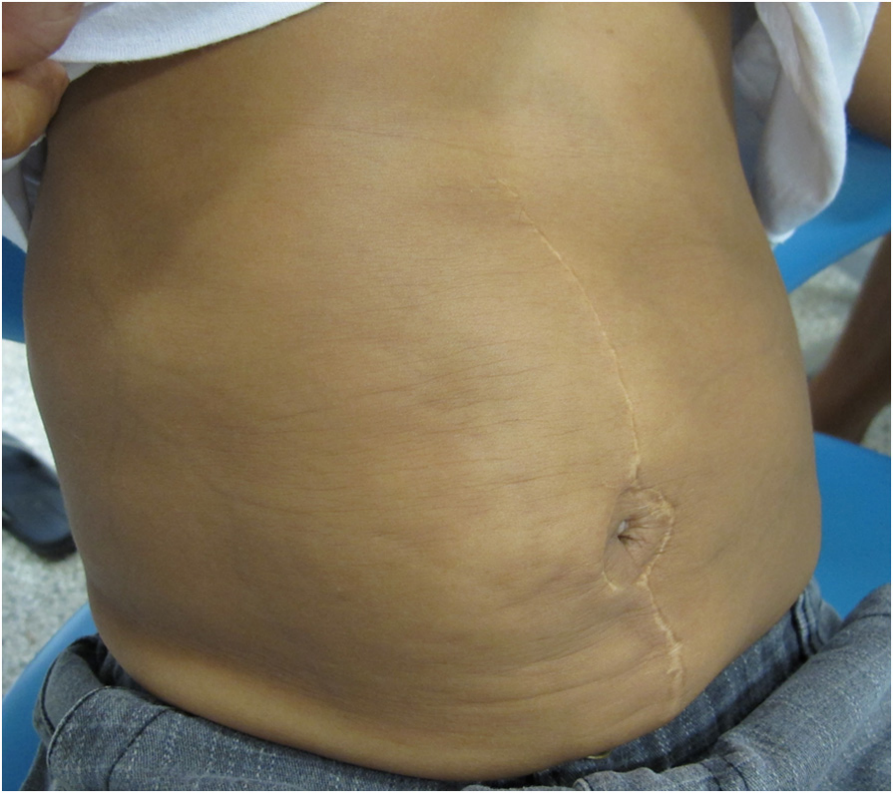
View of a distended and wrinkled abdomen.

The child underwent plastic surgery of the abdomen and penis and a surgery to position the testes correctly at 2 years of age. The present state of the child’s abdomen is shown in Figure 1.

**Figure 2:**
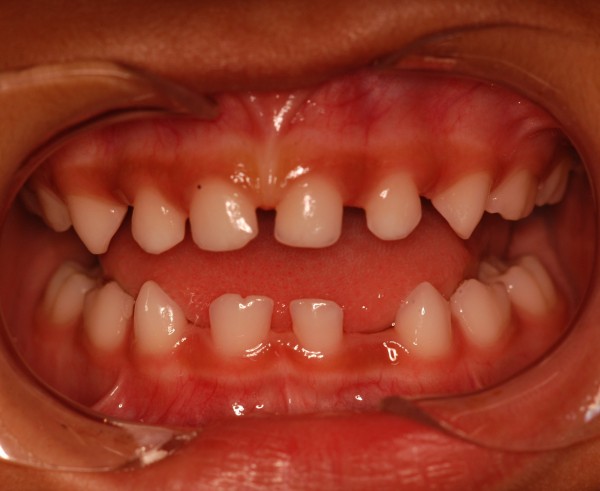
Intraoral view of the mandibular primary double right central incisor and congenitally absence of both right and left lateral primary incisors.

**Figure 3:**
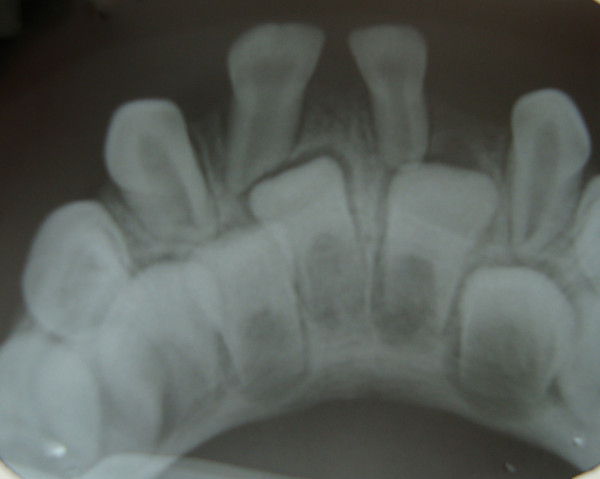
Periapical radiograph displaying a geminated mandibular right central primary incisor with one root and one root canal and absence of a mandibular permanent left incisor.

**Figure 4:**
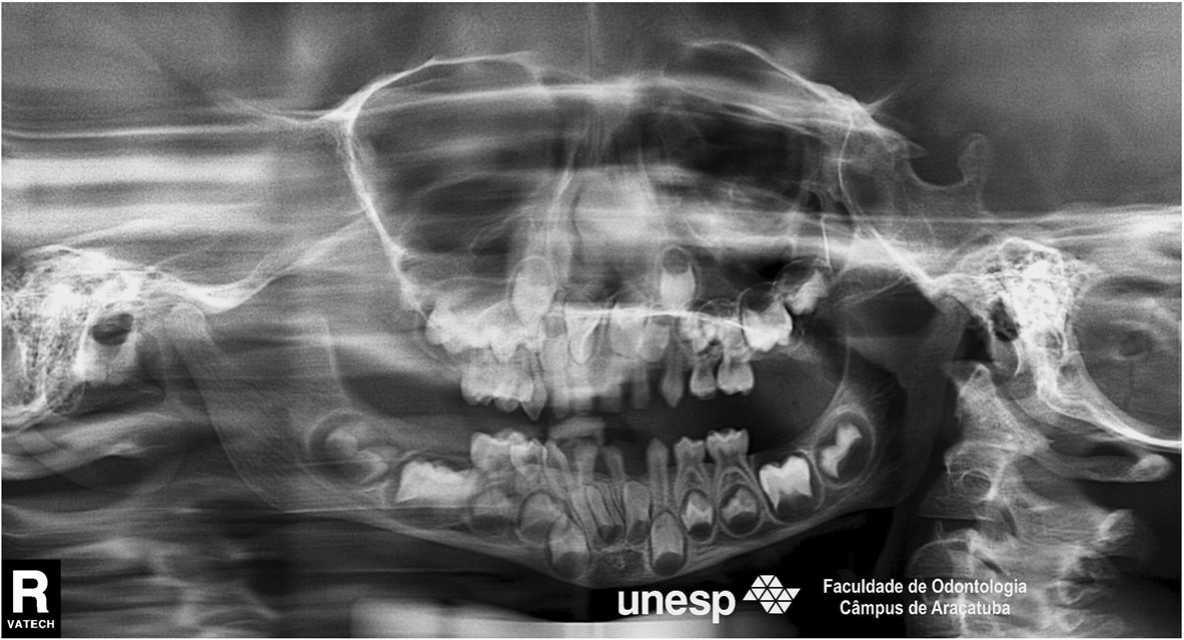
Orthopantomograph showing absence of a mandibular permanent incisor.

The following oral health status findings were observed at the initial clinical examination and in all follow-up appointments: no dental caries, low plaque indices, no supragingival calculus deposits, and no other periodontal problems. The following dental anomalies were clinically and radiographically noted when the primary dentition was complete: a geminated mandibular right central incisor with 1 root and 1 canal; the absence of a mandibular permanent left incisor (Figure 2 and 3); and congenitally missing primary teeth, namely, the mandibular right and left lateral incisors (Figure 2 and 3). In addition, agenesis of the mandibular left lateral permanent incisor germ was noted on radiography. All dental anomalies in the primary and permanent dentition were confirmed by panoramic radiography (Figure 4).

The child’s family received preventive and educational dental guidance and the child is seen by a dentist 4 times a year since his placement in the Baby Clinic of Araçatuba Dental School program. The parents have maintained strict oral health care of the child, which is demonstrated by the aspects cited above regarding the child’s oral health.

The monitoring system of the program and the parent’s access to appointments whenever necessary, i.e., not only in cases of dental emergency, allows the dental team to register any manifestation of oral diseases. Oral soft tissue manifestations arising from disorders caused by the syndrome [15-19] have not been noted up to this point.

Although metabolic disorders can affect mineralized structures [16], an alteration of dental enamel was not found in the primary dentition. However, it will be necessary to check for the presence of enamel hypoplasia in permanent teeth. A radiographic exam did not reveal bone problems [16].

While the etiology of PBS is unknown, the literature related to this disorder describes it as a mesodermal defect [4] consisting essentially of genitourinary tract abnormalities [2,3,6]. Nevertheless, the wide range of severities and the previously reported alterations of others organs [7,11,13], including the association with some syndromes [2,8,9], provide evidence that ectoderm tissues are affected by the syndrome [11,13]. This is the only report of a child with PBS associated with dental anomalies in literature. The dental anomalies found in this case report justify the necessity of an extra-abdominal assessment [13], especially an intra-oral evaluation. The importance of this anomaly tends to be underestimated because of the low prevalence of geminated teeth. However, anomalies in primary teeth can significantly affect the permanent successors teeth [20,21]. Several studies have shown that geminated primary teeth have an influence on permanent successors teeth, resulting in hypodontia (missing teeth), supernumerary teeth, repeated geminated teeth, and peg-shaped teeth [20-22]. In cases of geminated primary teeth involving the mandibular lateral incisors and canines, hypodontia of permanent successors is most common [20,21,23]. Careful monitoring is required, and orthodontic management should be considered part of a treatment plan to ensure functional occlusion and to advance esthetics.

## Conclusions

PBS has a broad spectrum of clinical features with different levels of severity, and the authors highlight the possibility that dental anomalies can be a part of this rare syndrome. The consensus is that treatment should be multidisciplinary. For maintenance of oral health, accurate intra-oral clinical and radiographic evaluations are required for patients with this syndrome in order to provide an early diagnosis of other abnormalities involving the primary and permanent dentitions.

### Consent

Written informed consent was obtained from the parents for publication of this Case report and any accompanying images. A copy of the written consent is available for review by the Series Editor of this journal.

## Competing interests

The authors declare that they have no competing interests.

## Authors’ contributions

MDB and COF participated in the clinical dental care of the patient and have continued performing regular clinical and radiographic follow-up. RFC supervised the clinical dental care of the patient. MDB was responsible for the literature search and wrote the paper. All authors read and approved the final manuscript.

## Pre-publication history

The pre-publication history for this paper can be accessed here:

http://www.biomedcentral.com/1472-6831/12/56/prepub
